# The Good and the Objectional Qualities of Oxyphosphate of Zinc as a Filling Material

**Published:** 1896-05

**Authors:** C. A. Quackenbush

**Affiliations:** U. of M.


### The Good and the Objectionable Qualities of Oxyphosphate of Zinc as a Filling Material.

BY C. A. QUACKENBUSH, U. OF M.

That we may be able to judge of the properties of a substance it is well to know what elements enter into its composition and under what conditions its formation is brought about.

Oxyphosphate of zinc contains three elements: oxygen, phosphorus and zinc. The chemical formula of a filling made of this material is $ZN\ (PO_3)_2$. It is the salt which results from the reaction of a solution of metaphosphoric, or glacial phosphoric acid on oxide of zinc. The reaction is as follows: $2\ HPO_3 + ZNO = ZN\ (PO_3)_2 + 2\ H_2O$. So that when a cavity is filled with oxyphosphate it is filled with a material which is at the moment in process of formation.

Now, in what degree does this new substance which is formed possess the qualities of an ideal filling material? We can best draw our conclusions as to the value of a material for filling teeth by determining how far it possesses those properties that must, to a greater or lesser extent, characterize every material used for that purpose. They are in order of preference: 1, indestructibility; 2, adaptability; 3, hardness; 4, cohesion; 5, non-conductivity; 6, tenacity; 7, color; and 8, the quality of being a non-irritant.

In discussing oxyphosphate we will notice that it partakes of some of those properties in a marked degree, while in others it is equally deficient.

Oxyphosphate is of all the materials used for permanent work the most destructible, unless we except oxychloride. It will withstand the assaults of neither chemical nor mechanical agents that are almost constantly at work in the mouth, and to which every exposed filling material is subjected for any considerable length of time—hardly long enough, indeed, to merit the name of permanent work, although it is often used for that purpose. The average life of a phosphate filling is from one to three years. In rare instances they have been known to have remained in good condition from ten to twenty years, while in many mouths they disintegrate in a few weeks or months.

In its adaptability it is almost, or quite, ideal. It can be readily and easily inserted into any cavity and does not require much, if any, retaining form to hold in position. For contouring, however, it is not very desirable, as it sets very rapidly, and contours made with it would be easily broken away. For filling under cuts where it is difficult to place gold properly, and where we do not wish to cut away more tooth substance in order to obliterate the undercut the phosphate is convenient and serviceable.

As to hardness, phosphate is far from being what it should be. In most cases it is soon worn away considerably in the process of mastication.

Its cohesive quality again places it in the front rank as a filling material. It is readily wrought into a solid mass. All of the particles cohere.

As a non-conductor of heat and cold it more than equals any of the other materials. And it is mainly this property, together with its cheapness and ease of adaptation, that gives to oxyphosphate its value as a filling material. Used as a foundation, especially with gold, it prevents largely the conduction of heat and cold to the pulp and protects it from irritation. It saves gold, saves time, saves labor and makes a cheaper and better filling than gold used alone.

It probably fails in tenacity as much as it excels in non-conductivity. It is easily chipped and broken away, which makes it objectionable where exposed to the occlusion of the teeth.

In its colors it attains nearer to perfection than any of the other plastics or of the metals, and is capable of being shaded by the addition of various substances, which perhaps do not aid in its durability.

In its non-irritant qualities it again excels. It is almost a non-irritant, owing to the phosphoric acid. On this account it is much more extensively used than oxychloride, which is irritant to a considerable degree.

To summarize : In its adaptability, cohesion, non-conductivity and non-irritant properties oxyphosphate is a good filling material. In its destructibility and lack of hardness and of tenacity it is not a good filling material. It has five good qualities to commend it, but is deficient in three important ones. Used in its place, it is one of the best of filling materials; but misused, it is one of the poorest.

